# Non-Enzymatic Browning of Collagen Hydrolysates with Chondroitin Sulfate Disaccharides from Turkey and Shark Cartilage

**DOI:** 10.3390/molecules31081241

**Published:** 2026-04-09

**Authors:** Anna Pudło

**Affiliations:** Department of Functional Food Products Development, Faculty of Biotechnology and Food Sciences, Wrocław University of Environmental and Life Sciences, Chelmonskiego Str. 37, 51-630 Wrocław, Poland; anna.pudlo@upwr.edu.pl

**Keywords:** Maillard reaction, collagen, chondroitin sulfate, browning, furosine, melanoidins, antioxidant activity

## Abstract

The aim of this study was to evaluate the feasibility of inducing non-enzymatic browning using enzymatic collagen hydrolysates from turkey knee cartilage and chondroitin sulfate disaccharides derived from turkey and shark cartilage. Glycation was carried out in aqueous solutions at 60–120 °C for 3 h. After glycation, furosine content and browning intensity were determined as indicators of intermediate and final Maillard reaction products. FTIR spectra, color parameters, and antioxidant properties were also analyzed. The results showed that chondroitin sulfate disaccharides were more reactive with collagen hydrolysates than glucose and produced glycation products with higher antioxidant activity. The sulfation site on the N-acetylgalactosamine residue linked to glucuronic acid influenced the characteristics of the Maillard reaction products, including higher antioxidant activity and increased redness in products derived from turkey chondroitin sulfate disaccharides compared with those derived from shark cartilage, despite very similar FTIR spectral characteristics.

## 1. Introduction

Until 1985, glycation was considered to encompass all reactions forming a sugar linkage with a protein or peptide, whether catalyzed enzymatically or not. Today, glycation refers exclusively to the non-enzymatic modification of proteins by saccharides [1]. This process corresponds to the so-called Maillard reaction, which results in the formation of characteristic brown products. These spontaneous chemical reactions begin with the formation of a covalent bond between the amino groups of proteins, peptides, or amino acids and the carbonyl groups of sugars [2,3]. During these reactions, various factors, including temperature, processing time, water activity, and pH, influence the formation of either colorless or colored Maillard reaction products (MRPs) at different stages [4].

The type and quantity of these products vary across the three classical main stages of the Maillard reaction. In the initial stage, Schiff bases are formed and subsequently rearranged into Amadori or Heyns products, which are colorless. The intermediate stage involves the breakdown of Amadori or Heyns products through a series of chemical processes including oxidation, dehydration, fragmentation, radical reactions, acid hydrolysis, enolization, Strecker degradation, and aldol condensation. In the final stage, extensive condensation and polymerization reactions lead to the formation of high-molecular-weight, brown-colored nitrogenous compounds known as melanoidins [2,5,6,7,8].

Non-enzymatic browning between basic food components (simple sugars and proteins) is relatively well understood despite their chemical complexity. The mechanism and kinetics of the Maillard reaction in model systems—including those simulating reactions occurring in food—are most commonly investigated using glucose as the substrate, although it is not the most reactive hexose [9]. Reactions between glucose and glycine, the predominant amino acid in collagen, have been extensively characterized. Glucosamine is also frequently used as a substrate because the presence of both an electrophilic carbonyl group and an amino group in its molecule leads to a distinct reaction pathway during the later stages of browning [10]. In addition, derivatives of simple sugars, such as uronic acids, have been examined as potential substrates. Bornik and Kroh [11] demonstrated that when D-galacturonic acid is used as the reducing sugar, the intensity of Maillard browning may be up to tenfold higher than that observed for pentoses or hexoses.

The outcomes of the Maillard reaction may include enhanced antioxidant activity in foods, more appealing aroma or color, as well as undesirable effects such as reduced nutritional value of proteins, food intolerances, and potential toxicity of MRPs [8,12,13]. Particularly strong antioxidant activity has been reported for MRPs derived from protein hydrolysates, which are currently recognized as an efficient approach for valorizing by-products generated during raw material processing, including those of animal origin [14]. Considerable attention has therefore been devoted to the full utilization of raw materials and by-products from animal carcass processing [15]. Bones and cartilage—such as those derived from poultry—remain largely underutilized despite being valuable sources not only of protein hydrolysates but also of polysaccharide components such as glycosaminoglycans (GAGs), including chondroitin sulfate (CS). These compounds form proteoglycans which, together with collagen, constitute the principal structural components of cartilaginous connective tissue [16].

CS is a polymer composed of repeating disaccharide units containing glucuronic acid and sulfated N-acetylgalactosamine (polymer of β-glucuronic acid-(1→3)-β-N-acetylgalactosamine-4-sulfate or -6-sulfate) [17]. Interactions between collagen and proteoglycans containing glycosaminoglycans play a key role in determining the structural integrity and mechanical properties of many tissues [18]. However, the presence of certain GAGs, particularly CS, in collagen solutions and in in vitro systems used to generate tissue constructs has been shown to reduce the mechanical properties of the resulting materials [19]. Lopez et al. [20] reported that enzymatic removal of GAGs using chondroitinase ABC—a bacterial lyase—from engineered tissue constructs resulted in improved mechanical strength.

Consequently, enzymatic depolymerization of CS has attracted increasing attention. One research direction focuses on the production of low-molecular-weight products, including disaccharides, which may exhibit enhanced biological activity [21]. Another application involves the enzymatic degradation of CS present in wastewater from the meat industry, where this polymer contributes to elevated chemical oxygen demand [22]. Current commercial approaches to CS degradation rely primarily on bacterial chondroitinases ABC belonging to the lyase class. These enzymes cleave β-1,4-glycosidic bonds between N-acetylgalactosamine residues and uronic acid residues via a β-elimination reaction, generating unsaturated uronic acid residues with a double bond between C4 and C5 (4,5-unsaturated hexuronate) [23].

Since meat-based hydrolysates typically contain only trace amounts of reducing sugars, the addition of reducing sugars or sugar-rich components is necessary to enhance the formation of MRPs and the associated flavor and aroma compounds. The enzymatic degradation of CS therefore produces unsaturated disaccharides that may serve as potential substrates for further chemical reactions. However, the ability of GAG-derived compounds—specifically the unsaturated disaccharides generated during CS depolymerization—to participate in Maillard reactions has not yet been investigated.

Previous studies have examined the reactions between animal protein hydrolysates, including collagen, and simple sugars or their derivatives [24,25]. However, the potential of GAGs or their components—specifically the disaccharide units forming the CS polymer—to participate in Maillard reactions remains unexplored. Interactions between complex polysaccharides such as GAGs (e.g., hyaluronic acid and chondroitin sulfate) or their components and proteins or their hydrolysates may influence the functional properties of foods or serve as a basis for developing carriers for bioactive compounds or drugs [26].

The degradation of GAGs by bacterial chondroitinases within the microbiota during digestion has recently emerged as a new research direction [27]. Moreover, the use of unsaturated CS degradation products has been suggested as a promising strategy for developing compounds with high antioxidant potential [28]. To date, knowledge of Maillard reactions between naturally occurring components of animal raw materials—such as collagen and GAGs or their derivatives—remains limited. Therefore, the aim of the present study was to characterize non-enzymatic browning products formed between enzymatic collagen hydrolysates and sugar substrates consisting of unsaturated disaccharides obtained through enzymatic degradation of CS by chondroitinase. The reactivity of these disaccharides was compared with that of glucose and glucosamine, which are commonly used in classical studies of Maillard reactions involving protein hydrolysates.

## 2. Results and Discussion

### 2.1. Characterization of Substrates

#### 2.1.1. GAGs—Chondroitin Sulfate

Chondroitin sulfate is the predominant glycosaminoglycan in the structure of proteoglycans in cartilage in animals. It is composed of disaccharide units that differ in the degree and position of sulfation, including non-sulfated chondroitin, chondroitin-4-sulfate, chondroitin-6-sulfate, chondroitin-2,6-disulfate, chondroitin-4,6-disulfate, chondroitin-2,4-disulfate, and trisulfated chondroitin [29]. In the present study, CS from turkey knee cartilage consisted primarily of chondroitin-4-sulfate (s4) at 52.7% and chondroitin-6-sulfate (s6) at 38.5%, while in shark cartilage, the corresponding values were 29.1% and 57.7%, respectively. The s4 and s6 proportions in turkey cartilage were close to those found in bovine CS, whereas the profile for shark cartilage aligned with values reported by multiple authors [29,30,31,32]. In the present study, CS disaccharides obtained by enzymatic depolymerization with chondroitinase ABC from turkey and shark cartilage served as the saccharide substrates.

#### 2.1.2. Collagen and Total Protein Content in Preparations

The lyophilized, reconstituted atelocollagen fibers obtained from turkey knee cartilage (ColTKC) using pepsin contained 56.1% total protein and 54.3% collagen. This indicates that ColTKC was highly pure, with collagen accounting for 96.7% of the total protein.

**Figure 1:**
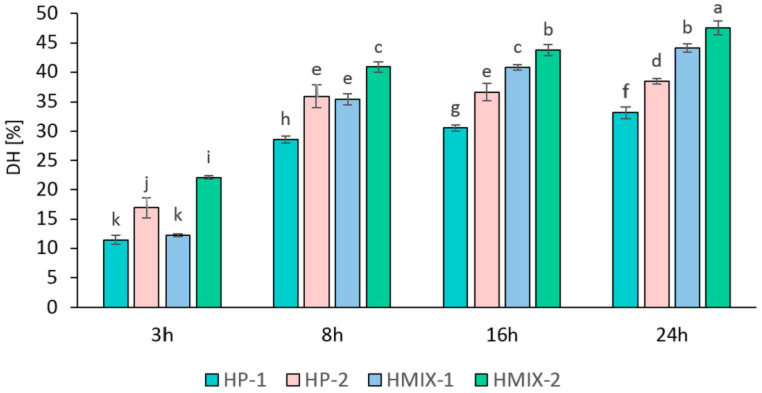
Degree of hydrolysis (DH) of turkey knee cartilage collagen hydrolyzed with Protamex (HP-1, HP-2) or with a cocktail of papain, Protamex, and Flavourzyme (HMIX-1, HMIX-2), depending on hydrolysis time (*n* = 3). Different letters indicate significant differences (*p* < 0.05).

For the production of hydrolysates rich in collagen peptides, enzymes such as papain, Alcalase, Protamex, Flavourzyme, and bromelain, which exhibit optimal activity at 50–60 °C, are most commonly used [33]. In the present study, it was established that increasing enzyme concentration and hydrolysis time significantly enhanced the degree of hydrolysis (DH) of collagen proteins (Figure 1). A marked increase in DH was observed after 8 h. Further extension of hydrolysis to 16 and 24 h resulted in slower yet statistically significant increases in DH compared to the 3–8 h interval.

The enzymatic hydrolysates obtained with the three-enzyme mixture (HMIX) reached a maximum DH of 44–47.5%, whereas the Protamex hydrolysate (HP) reached a maximum of 38.5%. Similar DH values (34–36%) for poultry-derived collagen were reported by Iltchenco et al. [34] after 2 h of bromelain and papain treatment, albeit with a high enzyme-to-substrate ratio of 8% (1:12.5). Likewise, Schmidt et al. [35] obtained 36% DH using Alcalase at a similar ratio. In contrast, with a lower Flavourzyme dosage (1:100 enzyme to substrate), Soladoye et al. [36] reported a maximum DH of 26% after 5 h. Considering that the maximum total enzyme addition in the present study was only 0.15% relative to the substrate, achieving a DH of 38–48% after 24 h may be considered highly efficient. This justified the selection of the HMIX and HP hydrolysates with the highest DH for subsequent analyses.

The total nitrogen content in the dry HP and HMIX hydrolysate preparations was 11.1% and 11.6%, respectively. The hydroxyproline content was 9.3% in HP and 8.4% in HMIX, corresponding to estimated collagen contents of 67.4% and 70.5%, respectively, based on hydroxyproline-to-collagen conversion factors derived from the total amino acid composition (Table 1). The free amino group content per gram of lyophilized sample was 24.0 mg/g for HP and 29.6 mg/g for HMIX.

#### 2.1.3. Amino Acid Profile of Atelocollagen and of the HP and HMIX Hydrolysates

The amino acid profiles of reconstituted atelocollagen fibers from turkey knee cartilage (ColTKC) and the enzymatic hydrolysates obtained with Protamex (HP) and the three-enzyme mixture (HMIX) are presented in Table 1. A key feature of both the collagen fibers and hydrolysates is the high glycine content, comprising 26–31% of the total identified amino acid residues. The combined content of proline and hydroxyproline—amino acids with a pyrrolidine ring—was as high as 23%. Hydroxyproline, a characteristic amino acid of collagen, is commonly used as a marker for collagen quantification. In the present study, hydroxyproline residues accounted for 13.2% of total amino acid residues in native collagen fibers (conversion factor to collagen: 7.56) and 11.9–13.7% in enzymatic hydrolysates. These values are close to the 13.8% reported by Ata et al. [37] for type II collagen isolated from chicken comb cartilage. In hydrolysates produced by thermal-enzymatic treatment with Protamex, the hydroxyproline level was close to that of native fibers. In contrast, the three-enzyme mixture treatment resulted in a lower hydroxyproline share (11.9%), consistent with the values reported by Cao and Xu [38] for collagen extracted from sternal and articular cartilage (11.7–12.1%), where glycine residues made up approximately 30% of the total.

### 2.2. Browning Detected at 360 nm and 420 nm

The concentration of intermediate water-soluble colored compounds can be assessed by measuring absorbance at 360 nm [39], whereas final reaction products, i.e., melanoidins, can be assessed by measuring absorbance at 420 nm in the visible spectrum [40].

**Figure 2:**
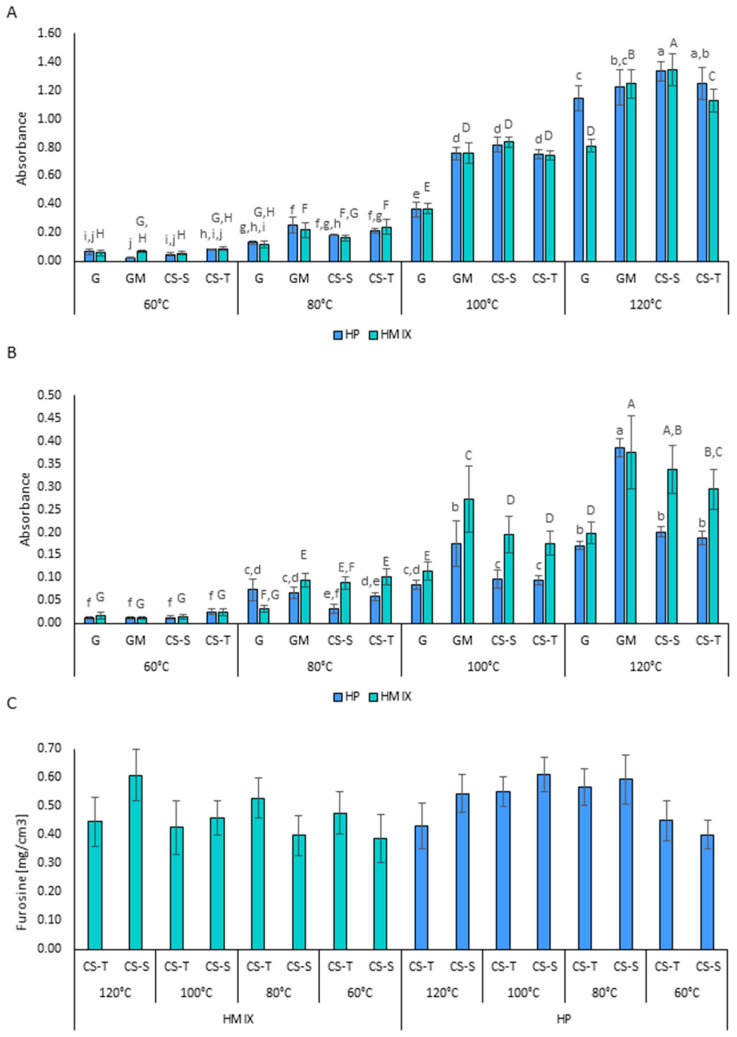
Maillard reaction products: (**A**) browning measured at 360 nm; (**B**) browning measured at 420 nm; (**C**) furosine content; (*n* = 3). HP—Protamex hydrolysate; HMIX—enzymatic hydrolysate obtained with the three-enzyme mixture (papain, Protamex and Flavourzyme); G—glucose; GM—glucosamine; CS-S—shark chondroitin sulfate hydrolysate; CS-T—turkey chondroitin sulfate hydrolysate. Capital letters indicate significant differences between samples for HMIX, lowercase letters for HP (*p* < 0.05).

In the present study, absorbance at 360 nm, indicating the quantity of intermediate Maillard reaction products, increased with incubation temperature—particularly above 80 °C—with significantly higher absorbance observed from 100 °C onward (Figure 2A). Only minor and inconsistent effects of the hydrolysate type on intermediate product formation were observed. A statistically significant difference was found only in the glucose (G) system at 120 °C, where absorbance at 360 nm was higher for the HP-G variant (Abs = 1.143) than for HMIX-G (Abs = 0.811).

The type of saccharide used in the reaction significantly affected the formation of intermediate Maillard reaction products. Absorbance at 360 nm was consistently higher in systems with glucosamine—even at 80 °C—compared to the glucose control (Figure 2A). No differences were noted between glucosamine (GM) and CS-derived disaccharides, except at 120 °C, where absorbance in CS-S disaccharide systems was higher than in other variants (HP: 1.335; HMIX: 1.345).

Absorbance at 420 nm, reflecting the degree of browning and the quantity of final melanoidins, followed trends similar to those observed at 360 nm. However, browning intensity in HP-based systems was often lower than in HMIX-based systems (Figure 2B). The highest absorbance at 420 nm was observed in glucosamine systems (except in the 120 °C HMIX-CS-S variant, where no significant difference from the GM system was found). Absorbance in the CS-based reactions was intermediate between that observed in the glucose and glucosamine systems. These results are consistent with literature data indicating that glucosamine is generally more reactive than glucose in Maillard reactions, resulting in greater quantities or intensities of browning products [41].

### 2.3. Furosine Level as Determined by High-Performance Liquid Chromatography

Furosine levels, used as a marker of early Maillard reaction products formed after Amadori rearrangement, showed similar levels across all systems containing shark- and turkey-derived CS disaccharides (Figure 2C). No clear relationship between furosine level and incubation temperature was observed, although a tendency toward elevated furosine levels was noted for HP disaccharide systems at 80 °C and 100 °C. The absence of a consistent relationship between furosine concentration and incubation temperature reflects fluctuations in the levels of this compound, formed from the reaction of the ε-amino group of lysine, as well as its depletion as this intermediate is consumed during the progression of the Maillard reaction. Nevertheless, the presence of furosine is recognized as a universal marker of thermal food processing [42].

The furosine levels observed in this study, ranging from 28 to 42 mg/100 g protein, were comparable to those reported by Mitra et al. [43], who indicated that such concentrations are indicative of thermal treatment of meat products, even at relatively low temperatures such as 58 °C.

### 2.4. Color of Dry Powders

Color parameters for the highly pigmented Maillard reaction products—resulting from the reaction of hydrolysates with CS-S and CS-T disaccharides, and GM—are presented in Table 2. In the case of Maillard reaction products with HP, a significant decrease in the lightness (L*) was observed as the reaction temperature increased from 60 °C to 120 °C, dropping from approximately 90 to 55. For HMIX-based products, L* values decreased from about 77 to the range of 30–40. These results align with the absorbance measurements at 360 nm and 420 nm and confirm the intensification of Maillard browning. The most pronounced darkening, as indicated by L* values, was observed in glucosamine-based reactions at 100 °C and 120 °C, corresponding with the highest absorbance values at 420 nm. However, Hong and Betti [44] reported that this highly reactive sugar does not significantly affect the L* parameter in solutions treated at lower temperatures (40 °C and 60 °C), which is consistent with the present results obtained at 60 °C for the HP and HMIX systems.

At 100 °C and 120 °C, reactions involving CS-S disaccharides, which are rich in 6-sulfated saccharides, showed greater darkening than reactions involving CS-T disaccharides, in which 4-sulfated saccharides predominated.

Some of the differences in browning between HP and HMIX hydrolysates—particularly in reactions with glucosamine—may be related to differences in amino acid composition. HMIX contained more alanine and histidine, despite a similar availability of glycine, which is known to strongly contribute to browning [45,46].

A significant increase in the red color component (a*) was also observed, rising from slightly negative values (indicating a slight blue tone) to over +7 at higher temperatures. At 60 °C and 80 °C, the a* parameter increased more in reactions containing CS-T disaccharides than in those containing CS-S. However, at temperatures ≥ 100 °C, a* values increased further in the CS-S systems. Systems containing glucosamine also exhibited high a* values, except in cases where the L* value had dropped substantially.

The increase in a* suggests that the formation of red pigments is a major pathway in Maillard reactions at elevated temperatures. Such pigments, derived from glucose and furan intermediates, are believed to include structures containing pyrrolopyrrole, pyrrole, and azepine rings [47,48,49]. Another important reaction pathway for hexoses and their derivatives is the formation of blue chromophores composed of two pyrrolopyrrole rings linked by a methine bridge [50,51]. These compounds may shift the a* value toward lower, or even negative, values. The observed decrease in a* for CS-T preparations at 80 °C and 100 °C (compared to 60 °C), which was not observed for CS-S systems, indicates that the sulfation position may influence the formation of colored Maillard reaction products.

Fluctuations in the yellow color component (b*) were generally smaller than those observed for a*, with most values oscillating around 20. Only in systems with glucosamine and CS-S disaccharides treated at 120 °C was a notable decrease in b* observed, likely due to the breakdown of intermediate yellow and orange pigments, which are ultimately converted into melanoidins at high temperatures. As shown by Frank and Hoffmann [52] and Murata [53], hexoses and disaccharides are capable of forming orange chromophores consisting of fused pyrrolidine and furan rings in glucose–proline reactions. Yellow pigments such as acetylpyridone and acetylazepinone—built around pyridone or azepinone rings—form in glucose–glycine systems [54]. Both of these amino acids (Gly and Pro), which are key to the formation of yellow and orange Maillard pigments, were present in large quantities in the collagen hydrolysates used in this study. This availability likely promoted the formation of stable pigments that helped maintain yellow color stability in the reaction products.

### 2.5. Antioxidant Properties

The antioxidant activity against ABTS^•+^ radicals of polar MRPs formed from Protamex hydrolysates with CS-S and CS-T disaccharides was higher than that of the untreated hydrolysates used as controls (Table 3). However, in reactions with glucose, the increase in ABTS activity was modest—rising from 25% to a maximum of 39% inhibition after treatment at 120 °C—compared to reactions involving other saccharides. In contrast, when glucosamine or CS-T disaccharides were used as substrates, the inhibition rate was significantly higher, reaching 60–80%, with peak activities also observed at 120 °C. At this highest temperature, strong radical scavenging activity was also observed in MRPs derived from CS-S disaccharides and HP hydrolysates. Similar trends were observed in the HMIX systems, although inhibition of ABTS radical formation in the HMIX–glucose system reached up to 50%, which was higher than that observed for the corresponding HP–glucose MRPs.

The ferric reducing antioxidant power (FRAP) generally increased with incubation temperature in all hydrolysate–saccharide systems (Table 3). However, in glucose systems, the increase in FRAP was less pronounced compared to those with other saccharides, which already showed high activity at 60 °C. This trend was consistent across both types of hydrolysates tested. A particularly important observation was the exceptionally high antioxidant activity of MRPs derived from glucosamine, which not only exceeded that of glucose-derived MRPs but also frequently surpassed those of CS-based products. This property of glucosamine is well documented in the literature [55] and has been attributed to its strong ferrous ion-chelating ability and its capacity to protect proteins against hydroxyl radical-induced oxidation.

The antioxidant activity of CS-derived MRPs—often comparable to glucosamine MRPs—varied by assay type (ABTS vs. FRAP) and CS origin. In general, disaccharides derived from turkey cartilage, which are rich in 4-sulfated N-acetyl-D-galactosamine (GalNAc-4S), showed higher antioxidant activity than shark-derived disaccharides, in which sulfation occurs predominantly at position 6 [56]. Campo et al. [57] similarly reported stronger antioxidant properties for GAGs sulfated at position 4, with minimal effects from those sulfated at position 6.

### 2.6. Principal Component Analysis (PCA)

Principal component analysis was applied to determine the relationships between the conditions of the non-enzymatic browning process (temperature, collagen hydrolysate variant, type of saccharide) and the amount of colored compounds measured spectrophotometrically at 360 and 420 nm, color parameters (L*, a*, b*), and antioxidant characteristics (ABTS and FRAP) of the resulting products. The first two components jointly accounted for 86.76% of the variance in the original variables (PC1 explained 72.16% and PC2 14.57%).

**Figure 3:**
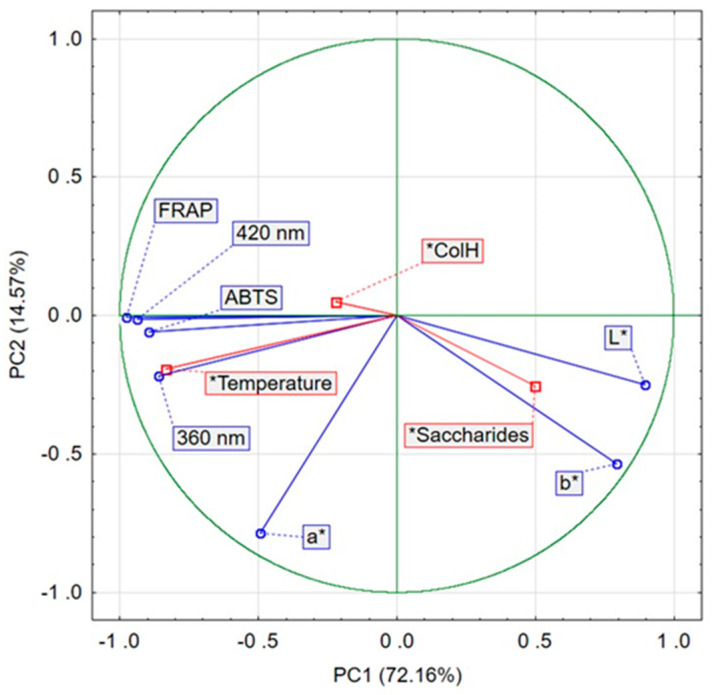
Principal Component Analysis (PCA) of the conditions of the non-enzymatic browning process, the colorimetric and antioxidant characteristics of the resulting MRPs. PC1—principal component 1; PC2—principal component 2.

According to the PCA results, temperature was the dominant factor affecting both the course of the Maillard reaction and the characteristics of the MRPs formed in this experiment (Figure 3). The saccharide type showed a weaker, yet noticeable influence, while the collagen hydrolysate variant (ColH) exerted only a minor effect, as indicated by the short lengths of its variable vectors. The formation of intermediate MRPs (360 nm) and final reaction products (420 nm) was strongly and positively correlated with temperature (0.97 and 0.87, respectively).

The antioxidant activity (ABTS and FRAP) of the non-enzymatic browning products was highest in samples containing high concentrations of final reaction products measured at 420 nm. In contrast, the L* (lightness) and b* color parameters showed an opposite pattern: they were the only variables that showed strong positive associations with saccharide type, while displaying negative correlations with the remaining variables.

### 2.7. FTIR Spectra Analysis

**Figure 4:**
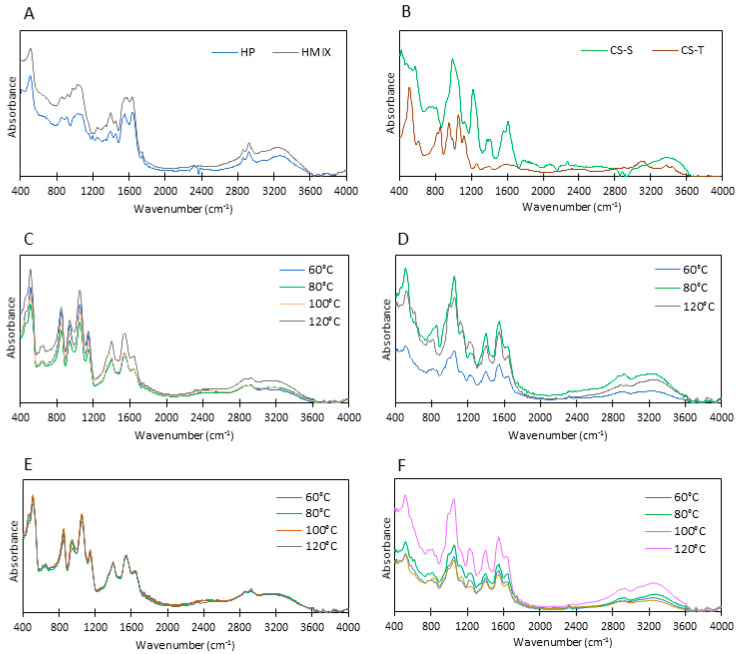
FTIR spectra of collagen hydrolysates (**A**), chondroitin sulfate (**B**) and Maillard reaction products (**C**–**F**): (**C**) HP/CS-T; (**D**) HP/CS-S; (**E**) HMIX/CS-T; (**F**) HMIX/CS-S. HP—Protamex hydrolysate; HMIX—enzymatic hydrolysate obtained with the three-enzyme mixture (papain, Protamex and Flavourzyme); CS-S—shark chondroitin sulfate; CS-T—turkey chondroitin sulfate.

The FTIR spectra of HP and HMIX hydrolysates, turkey and shark cartilage glycosaminoglycans, and the products of collagen hydrolysate glycation with CS-S and CS-T disaccharides are shown in Figure 4A–F. Bands below 600 cm^−1^ were detected at 506 cm^−1^ (HP) and 525 cm^−1^ (HMIX) for the collagen hydrolysates (Figure 4A). These bands correspond to the mode at 498 cm^−1^ described by Krimm and Bandekar [58], which has been attributed to skeletal deformations of peptide molecules (mainly C–C–N interactions) and commonly referred to as amide VI. In the spectra of shark CS disaccharides, the most intense band appeared at 423 cm^−1^, accompanied by a weaker band at 577 cm^−1^, while turkey CS displayed a pronounced band at 509 cm^−1^ (Figure 4B). These signals are associated with skeletal vibrations and hydrogen bonding in saccharide molecules [59]. In the glycation products, bands were observed at 503–508 cm^−1^ for CS-T and 514–537 cm^−1^ for CS-S (Figure 4C–F). In the next spectral region (600–660 cm^−1^), bands were recorded at 651–657 cm^−1^ for the hydrolysates, at 611–634 cm^−1^ for the glycation products with CS-T, and at 589–623 cm^−1^ for those with CS-S. Qi et al. [25] reported a decrease in absorbance near 660 cm^−1^ in FTIR spectra after glycation of bone hydrolysates, attributing this effect to the involvement of NH_2_ groups in the Maillard reaction, which reduces N–H bending vibrations. In this study, such a decrease was observed only in the spectrum of the HMIX glycation products, which may be related to their higher degree of hydrolysis, and, consequently, the greater availability of NH_2_ groups compared with HP.

The subsequent low-wavenumber regions are present in the spectra of protein hydrolysates but are particularly characteristic of carbohydrates. In their classic study, Mathlouthi and Koenig [59] noted that the 1200–700 cm^−1^ region constitutes the carbohydrate fingerprint, although in many cases this range narrows to 950–750 cm^−1^. Within this part of the spectrum, in sugars containing sulfate esters (including the disaccharides that constitute CS) and in their reaction products with peptides, a key segment is the 800–860 cm^−1^ range, which contains bands associated with –C–O–S interactions [60]. Clear differences occur between the spectra of CS sulfated at C4 of N-acetylgalactosamine (s4—predominant in avian CS, including turkey) and those sulfated at C6 (s6—predominant in shark cartilage). For CS with dominant s4, Garnjanagoonchorn et al. [61] and Myron et al. [62] reported maximum absorbance near 850–857 cm^−1^, whereas for CS dominated by s6, maxima occur around 820–824 cm^−1^. In the spectra of shark CS disaccharides obtained in this study, a broad band with three weakly defined bands was observed between 737–811 cm^−1^, while the spectrum of turkey CS disaccharides displayed a distinct band at 857 cm^−1^, characteristic of C–O–S interactions typical of s4-CS. Similarly, the absorbance maxima of the reaction products of collagen hydrolysates with CS-T (dominated by s4) appeared at 851–857 cm^−1^, whereas those for CS-S products were distributed across a broader range, 794–845 cm^−1^, and exhibited lower intensity (Figure 4D,F). The increased absorbance in the 851–857 cm^−1^ region for CS-T products may indicate only partial utilization of the available CS disaccharides in the Maillard reaction. The observed differences between the two disaccharide types (s4 and s6) suggest that s6 sugars may interact more readily with proteins or peptides than s4 sugars.

When interpreting spectra in the 900(880)–1200 cm^−1^ region, it is necessary to account for features characteristic of collagen hydrolysates, including maximum bands at 1051 cm^−1^ for HP and 1063 cm^−1^ for HMIX (Figure 4A). These bands exhibit lower absorbance than those observed in the IR spectra of the glycation products with CS-T and CS-S (Figure 4C–F). This region is described in the literature as the carbohydrate fingerprint zone of the IR spectrum [59], reflecting skeletal C–H and C–OH deformation modes, yet it is also typical of peptides owing to N–C skeletal stretching [58]. For sugars, the fingerprint region contains the most prominent IR bands resulting from saccharide ring deformations [63], and, as noted by Kacurakova and Mathlouthi [64], it additionally includes bands indicative of glycosidic bonds (band near 966 cm^−1^). In the CS-T disaccharide spectra, two distinct bands appeared at 943 cm^−1^ (with a shoulder at 983 cm^−1^) and a dominant band at 1057 cm^−1^ (with a shoulder at 1120 cm^−1^) (Figure 4B). The CS-S disaccharide spectrum differed slightly and was dominated by a single band at 989 cm^−1^ (with a left-side shoulder at 943 cm^−1^ and a right-side shoulder at 1063 cm^−1^) and a weak band at 1120 cm^−1^ (Figure 4B).

The IR spectra of the glycation products of collagen hydrolysates displayed three bands in the 900–1200 cm^−1^ region. In samples glycated with turkey cartilage CS disaccharides dominated by s4, these bands appeared clearly separated at approximately 1150 cm^−1^, around 1060 cm^−1^ (the highest band), and near 950 cm^−1^ (Figure 4C,E). In spectra from shark cartilage (with predominant s6), the highest band (1040–1060 cm^−1^) was well defined, while the remaining two typically formed shoulders flanking it (at 971–1005 cm^−1^ and 1108–1148 cm^−1^) (Figure 4D,F). The dominant 1040–1060 cm^−1^ band corresponds to the major band commonly observed in sugar spectra near 1050 cm^−1^ [65,66], and, as reported by Rozenberg et al. [67], a strong absorbance band in this region is also a characteristic marker of protein glycation.

The band near 1150 cm^−1^ may be attributed to glucuronic acid [66], a structural component of CS disaccharides. In sugar–protein systems, however, this band may also reflect Maillard reaction progression. Yang et al. [68] report that a band near 1180 cm^−1^, arising from C–O–C and C–O stretching, is characteristic of melanoidin frameworks containing carbohydrate side chains. As in the present study, Qi et al. [25] associate increased absorbance in the 1000–1200 cm^−1^ region with the attachment of saccharides to collagen hydrolysate peptides.

The next IR spectral region, 1200–1500 cm^−1^, in amino acids is associated with Amide III–type vibrations [69,70], arising from C–N stretching and N–H bending. In polypeptides, the characteristics of these modes depend on the structure of the side chains, which leads to the appearance of multiple bands between 1400 and 1200 cm^−1^ [71]. In the spectra of collagen hydrolysates, two weak bands were identified at approximately 1380 cm^−1^ and 1430 cm^−1^ (Figure 4A). In CS-S disaccharide spectra, a strong band occurred at 1217 cm^−1^ along with two weak signals between 1371–1417 cm^−1^, whereas CS-T spectra showed only very faint features in this region (Figure 4B).

Within the 1200–1500 cm^−1^ range, the observed bands correspond to symmetric CH_2_ deformation and numerous C–OH deformation modes typical of carbohydrates [59]. In the present study, the 1200–1400 cm^−1^ region was more pronounced in shark CS spectra than in turkey-cartilage CS, consistent with the observations of Garnjanagoonchorn et al. [61]. A clear band at 1251 cm^−1^ was also noted in the spectra of hydrolysates glycated with CS-S disaccharides dominated by s6 (Figure 4D,F). As reported by Vergne et al. [24], this band is characteristic of collagen glycation, and the increase in absorbance reflects a higher number of C–N groups formed during glycation [68]. In the spectra of the glycation products, a distinct band appeared near 1400 cm^−1^ (1380–1411 cm^−1^) (Figure 4C–F), with higher absorbance than in the spectra of the hydrolysates (Figure 4A). The enhanced intensity in this region may arise not only from sugar–peptide interactions but also from characteristic bands of CS disaccharide constituents, including N-acetylgalactosamine [65] and glucuronic acid, both associated with CCH and COH bending modes [66].

Bands corresponding to the amide II region (C–N–H bending) in the FTIR spectra of collagen, its hydrolysates, or gelatin have been reported at several wavenumbers, including 1540 cm^−1^ [69], 1560 cm^−1^ [70], and 1544 cm^−1^ [72]. In this study, the amide II bands in the hydrolysate spectra appeared at 1565 cm^−1^ for HMIX and 1537 cm^−1^ for HP. Following glycation, they were observed at 1542–1554 cm^−1^, accompanied by a marked increase in absorbance relative to the hydrolysates for both CS-T and CS-S. Ioannou and Varotsis [73] reported a similar increase in absorbance at 1575 cm^−1^ during the reaction between a reducing sugar and an amino acid, attributed to the formation of a Schiff base.

In the present study, bands in the 1611–1645 cm^−1^ range, corresponding to amide I transitions (C=O stretching vibrations) [69], decreased after the reaction of the hydrolysates with CS disaccharides. Comparable reductions in absorbance after glycation have been described by Vergne et al. [24], Yang et al. [68], and Qi et al. [25] in studies involving collagen, hydrolysates, and amino acids. The wavenumber of the maximum band in the amide I region (1640–1645 cm^−1^) for the hydrolysates examined is close to the value reported by Barth [71] for α-helical secondary structures. These structures may be preserved to a limited extent because lyophilized hydrolysates still contain a significant amount of protein molecules and large peptides. This pattern contrasts with that observed in other IR regions, where the spectral changes suggest stronger interactions between CS-S disaccharides and collagen hydrolysate peptides than between CS-T disaccharides and these peptides.

## 3. Materials and Methods

### 3.1. Reagents

The following enzymes were used in the study: pepsin from porcine gastric mucosa (≥400 U/mg protein), papain from papaya latex (1.5–10 U/mg solid), and chondroitinase ABC from *Proteus vulgaris* (50–250 U/mg protein), all purchased from Merck (Darmstadt, Germany). Protamex^®^ (>1.5 AU-N/g) and Flavourzyme^®^ (≥500 U/g) were obtained from Novonesis (Kongens Lyngby, Denmark).

The following chemical reagents and standards were used: D(+)-glucosamine hydrochloride (≥99%, Merck, Darmstadt, Germany), chondroitin sulfate sodium salt from shark cartilage, sodium 1-heptanesulfonate, hydroxyproline, TNBS (2,4,6-trinitrobenzenesulfonic acid solution), 4-(dimethylamino)benzaldehyde (98%), ABTS (2,2′-azino-bis(3-ethylbenzothiazoline-6-sulfonic acid) diammonium salt; ≥98%), glycine (≥99%), TPTZ (2,4,6-tris(2-pyridyl)-s-triazine, ≥98%), absolute ethanol, guanidine hydrochloride (≥99%), acetonitrile for HPLC (≥99.9%), and formic acid, all from Merck (Darmstadt, Germany). Furosine (2-furoylmethyl-lysine) was purchased from PolyPeptide (Strasbourg, France). Additional reagents used during the study included glucose, chloramine-T, phosphate buffers, tris(hydroxymethyl)aminomethane, hydrochloric acid, trichloroacetic acid (Chempur, Piekary Śląskie, Poland), sodium acetate (EUROCHEM BGD Sp. z o.o., Tarnów, Poland), and citric acid (P.P.H. STANLAB Sp. z o.o., Lublin, Poland), all of analytical grade.

### 3.2. Materials

Two types of raw materials were used in the study. Reconstituted atelocollagen fibers (ColTKC), used as the source of protein hydrolysates, were obtained from turkey knee cartilage using the enzymatic method described by Pudło et al. [74]. Collagen extraction was carried out using pepsin in a citric acid solution at pH 2.0 and 20 °C. The collagen fibers were then reconstituted using sodium chloride (final concentration 0.9 M), followed by two-stage dialysis (first in distilled water, then in 0.01 M phosphate buffer at pH 7.0). The resulting collagen fibers were lyophilized.

The carbohydrate source consisted of animal-derived polysaccharides, specifically chondroitin sulfate, a GAGs isolated from turkey knee cartilage using a modified method based on Luo et al. [75]. In the first step, proteoglycans were extracted using a 3.0 M guanidine hydrochloride solution (24 h, 20 °C). In the second step, GAGs chains were separated from proteoglycans structures using papain (60 °C, 24 h, phosphate buffer pH 7.0). The chondroitin sulfate fraction was then precipitated from the solution using ethanol in a 1:2 (*v*/*v*) ratio, and the resulting precipitate was lyophilized.

### 3.3. Experimental Procedure

#### 3.3.1. Collagen Protein Hydrolysis

In the first stage of the study, reconstituted native collagen from turkey knee cartilage was subjected to enzymatic hydrolysis under conditions that also promoted thermal denaturation. Proteolytic enzymes with optimal activity at approximately 60 °C were used, i.e., above the collagen denaturation temperature. The hydrolysis process was optimized with respect to the enzyme composition, incubation time, and enzyme concentration. Protamex was used at 5 mg/g (HP-1) and at 10 mg/g (HP-2) of raw material, with hydrolysis conducted at 60 °C for 3–24 h. Enzyme mixtures composed of papain, Protamex, and Flavourzyme were also tested (HMIX-1 at 2.5 mg/g and HMIX-2 at 5.0 mg/g of each enzyme per gram of raw material; 60 °C, 3 h to 24 h). Hydrolysis was carried out in an aqueous environment buffered with 0.01 M sodium phosphate at pH 7.0. After the process, enzyme inactivation was performed at approximately 100 °C for 20 min. Undigested collagen was removed by centrifugation (5200× *g*, 15 min, 4 °C) (MPW-351R, MPW MED. INSTRUMENTS, Warsaw, Poland). The hydrolysate solutions obtained from turkey knee cartilage collagen that showed the highest degree of hydrolysis, one from the HP group and one from the HMIX group, were lyophilized (FreeZone^®^ freeze dryer, Labconco Corp., Kansas City, MO, USA) and used in subsequent experiments.

#### 3.3.2. Glycosaminoglycan Degradation

In the second stage, chondroitin sulfate from turkey knee cartilage (CS-T) and shark cartilage (CS-S) was subjected to enzymatic degradation using chondroitinase ABC (reaction medium: 33 mM Tris-HCl and 33 mM sodium acetate, pH 8.0, incubation at 37 °C). After 24 h of incubation, the enzyme was inactivated at approximately 100 °C for 20 min, and the CS solutions were then lyophilized. Complete degradation of CS to disaccharide units was verified using the method described by Farndale et al. [76], by determining the presence of intact CS polymer in the reaction solution before and after treatment with chondroitinase ABC.

#### 3.3.3. Glycation Reaction

In the final stage of the study, Maillard reactions were initiated in model systems consisting of hydrolysates of reconstituted atelocollagen fibers and degradation products of chondroitin sulfate. Reference and control reactions were also conducted using glucose and glucosamine. Non-enzymatic browning reactions were carried out in aqueous solutions at temperatures ranging from 60 °C to 120 °C. Based on preliminary optimization, the reaction time was set at 3 h, which was sufficient to induce measurable color changes even at the lowest temperatures while avoiding complete blackening at the highest temperatures.

Because Carabasa-Giribet and Ibarz-Ribas [77] and Echavarría et al. [13] reported that an excess of reducing sugar relative to amino nitrogen shortens the induction period of Maillard product formation, a 3:1 molar ratio of sugars to available NH_2_ groups was applied in the present study. This approach is supported by Naranjo et al. [78], who noted that such an excess increases the concentration of open-chain sugar forms, which are more reactive with amino nitrogen in the Maillard reaction.

Accordingly, the Maillard reactions were conducted for 3 h in aqueous solutions prepared by dissolving or rehydrating the components and containing 0.033 M amino groups and 0.1 M sugars, including glucose, glucosamine, and CS disaccharides. Each experimental variant thus maintained a molar ratio of free NH_2_ groups to reducing sugar of 1:3. Measurements of browning intensity and furosine content were carried out using the reaction solutions, while the remaining analyses were performed on preparations obtained by lyophilizing the post-reaction solutions.

### 3.4. Total Nitrogen and Protein Analysis

The pure collagen preparation as well as the HP and HMIX hydrolysates were analyzed for total nitrogen and protein content using the AOAC method [79].

### 3.5. Degree of Hydrolysis

The degree of hydrolysis (DH) was determined based on the method described by Ovissipour et al. [80] with modifications. Following enzymatic hydrolysis, the hydrolysate was mixed with an equal volume (1:1, *v*/*v*) of 30% trichloroacetic acid (TCA). After 16 h of incubation at room temperature, the mixture was centrifuged at 12,000× *g* for 15 min at 18 °C (MPW-351R, MPW MED. INSTRUMENTS, Warsaw, Poland). The nitrogen concentration in the resulting supernatant was determined by the Kjeldahl method. The degree of hydrolysis was calculated using the following formula:

DH (%) = (CH/CS) × 100

where

CH—nitrogen concentration in the sample after precipitation with 15% TCA;

CS—nitrogen concentration in collagen prior to enzymatic hydrolysis.

### 3.6. Free Amino Groups

The concentration of free amino groups in the collagen hydrolysate preparations after rehydration was determined spectrophotometrically (Thermo Fisher Scientific Inc. Evolution 160 UV-Vis, Waltham, MA, USA) using TNBS (2,4,6-trinitrobenzenesulfonic acid) reagent, as described by Kuchroo et al. [81]. The measurements were based on standard solutions of glycine, with concentrations converted to amino group equivalents. Results were expressed as the amount of free NH_2_ groups (mg) per gram of lyophilized hydrolysate prior to rehydration.

### 3.7. Amino Acid Profile Including Hydroxyproline

The amino acid profile in collagen and hydrolysates (HP, HMIX) was determined according to AOAC Method 982.30, following acid hydrolysis in 6 M hydrochloric acid (24 h, 110 °C). Separation and identification of individual amino acids were performed using an AAA-400 amino acid analyzer (INGOS, Prague, Czech Republic).

Hydroxyproline (HPro) content was determined following ISO 3496:1994(E) [82]. The samples were hydrolyzed in 3 M sulfuric acid (H_2_SO_4_) at 105 °C for 16 h, followed by a two-step colorimetric reaction using:

(i) a chloramine-T solution (1.41 g sodium N-chloro-p-toluenesulfonamide trihydrate in 100 cm^3^ of pH 6.8 buffer);
(ii) a color reagent (10 g p-dimethylaminobenzaldehyde in 35 cm^3^ of 60% perchloric acid and 65 cm^3^ of 2-propanol).

Absorbance was measured at 558 nm using an Evolution 160 UV-Vis spectrophotometer (Thermo Fisher Scientific Inc., Waltham, MA, USA). The calibration curve was prepared using hydroxyproline standards in the range of 0–1.5 µg/cm^3^.

### 3.8. Analysis of Chondroitin-4-Sulfate and Chondroitin-6-Sulfate Proportions

The analysis was conducted according to a modified version of the method used by Volpi [30]. CS-T and CS-S samples were digested with chondroitinase ABC at a dose of 0.2 U/10 mg GAG (37 °C, 24 h). Disaccharide separation was performed via chromatography using SAX-HPLC (Spherisorb^®^; 5 µm; 4.6 × 150 mm; Waters Corp., Milford, MA, USA) with a flow rate of 1 cm^3^/min and detection at 232 nm. Mobile phase A contained 50 mM NaCl (pH 4.0), and phase B contained 1.2 M NaCl (pH 4.0). Gradient elution was performed from 5% to 90% phase B over 60 min.

### 3.9. Analysis of Non-Enzymatic Browning Reaction Products

#### 3.9.1. Furosine Content—Initial Stage of the Maillard Reaction

Furosine quantification was performed according to a modified version of the method used by Delgado-Andrade et al. [39], using high-performance liquid chromatography with a 1220 Infinity LC system and diode array detector (Agilent Technologies Inc., Santa Clara, CA, USA). The separation was conducted on a ZORBAX SB-C18 column (5 µm, 4.6 × 150 mm; Agilent Technologies Inc., Santa Clara, CA, USA) at 35 °C. The isocratic mobile phase consisted of 5 mM sodium heptanesulfonate, 20% acetonitrile, and 0.2% formic acid, with a flow rate of 0.7 cm^3^/min and an injection volume of 40 µL. Detection was carried out at 280 nm. The calibration curve was established using furosine standards in the range of 10–100 µg/cm^3^, and results were expressed relative to the protein content of the sample.

#### 3.9.2. Browning—Intermediate and Final Maillard Reaction Products

The progression of Maillard browning was assessed according to a modified version of the method described by Kang [83], by analyzing both intermediate products and final melanoidins. Absorbance of the solutions after glycation was measured at wavelengths of 360 nm and 420 nm using a UV-Vis spectrophotometer (Evolution 160, Thermo Fisher Scientific Inc., Waltham, MA, USA). Solutions were centrifuged at 12,000× *g* for 15 min at 5 °C (MPW-351R, MPW MED. INSTRUMENTS, Warsaw, Poland). The resulting supernatants were diluted 10-fold to keep absorbance values below 1.500 [12].

### 3.10. Fourier Transform Infrared Spectroscopy (FTIR)

FTIR analysis was conducted using a Fourier transform infrared spectrometer (IRSpirit, Shimadzu Corp., Kyoto, Japan). For each sample, the spectrum represented the average of 32 scans collected in the range of 4000–400 cm^−1^ at a resolution of 4 cm^−1^. The analysis was performed on dry, lyophilized Maillard reaction products.

### 3.11. Color of Powders

The color of the dried hydrolysates and Maillard reaction products was evaluated using a Konica Minolta CR 400 colorimeter (Konica Minolta, Inc., Tokyo, Japan) in the international CIE color system: L* (lightness), a* (redness (+)/greenness (−)), and b* (yellowness (+)/blueness (−)). A standard white calibration plate (Y = 93.8; x = 0.3133; y = 0.3195) was used as a reference.

### 3.12. Antioxidant Properties

The antioxidant activity of the hydrolysates and Maillard reaction products was assessed by spectrophotometric methods using a UV-Vis spectrophotometer (Evolution 160, Thermo Fisher Scientific Inc., Waltham, MA, USA):

○ ABTS radical scavenging activity was measured according to the method of Re et al. [84], using ABTS radicals diluted in PBS (pH 7.4); this assay primarily detects antioxidant activity of polar compounds.
○ Ferric reducing antioxidant power (FRAP) was determined according to Benzie and Strain [85].

The FRAP results were expressed as mM Fe^2+^ per gram of dry matter of Maillard reaction products. ABTS antioxidant activities were expressed as percentage inhibition (IHB), calculated using the formula

IHB (%) = ((Abs0 − AbsS)/Abs0) × 100

where

Abs0—absorbance of the ABTS solution before sample addition;

AbsS—absorbance of the ABTS solution after incubation with the sample.

### 3.13. Statistical Analysis

The results were subjected to analysis of variance (ANOVA) and principal component analysis (PCA) using STATISTICA 13.3 software (TIBCO Software Inc., Palo Alto, CA, USA). Duncan’s test (*p* ≤ 0.05) was applied to determine significant differences between means. Mean values from three replicates (*n* = 3) and the corresponding standard deviations (±SD) are presented in tables and figures.

## 4. Conclusions

The study demonstrated that disaccharides derived from chondroitin sulfate in turkey and shark cartilage exhibited higher reactivity than glucose in non-enzymatic browning reactions with collagen hydrolysates. The browning intensity of solutions containing CS disaccharides was generally lower than that observed in glucosamine-containing systems, although at 120 °C the values were comparable. This was consistent with the antioxidant activity of the products formed with CS disaccharides, which was higher than that of Maillard reaction products formed with glucose.

The origin of the GAGs and the sulfation position of N-acetylgalactosamine (C4 vs. C6) appeared to influence selected characteristics of the Maillard reaction products formed with collagen hydrolysates, particularly in color changes, such as the increased intensity of red pigments in reactions with CS-S disaccharides as temperature increased, whereas this effect was reduced in CS-T disaccharide systems. The products formed from collagen hydrolysates and CS-T disaccharides also showed higher antioxidant activity than those obtained with CS-S disaccharides. Minor differences were also observed in the FTIR spectra of the Maillard reaction products, such as stronger interactions in the amide III region (N-H bending) in products containing shark-derived disaccharides, a pattern not observed for turkey-derived disaccharides.

Overall, the Maillard reaction mechanism appeared to be similar for both types of CS disaccharides. This interpretation is supported by FTIR spectral changes observed in products formed with both types of disaccharides, which showed typical modifications after glycation of collagen hydrolysates, including reduced signals in the amide I and amide II regions. 

The conducted studies did not clearly demonstrate the influence of the sulfation site of N-acetylgalactosamine on the properties of the Maillard reaction products. This is because the experiments used CS depolymerization products that were chemically heterogeneous and differed in their sulfation patterns. In shark CS, 6-O-sulfation predominates, whereas in turkey CS the dominant form is 4-O-sulfation.

This issue is particularly relevant for saccharide units containing unsaturated uronic acid residues, which are increasingly generated during the enzymatic depolymerization of GAGs. Their increasing occurrence is associated with the growing commercial use of bacterial lyases for controlled modification of GAG molecular mass. As enzymatic depolymerization of GAGs becomes more widely applied in the processing of animal as well as plant-derived materials, especially marine materials, both partial and extensive degradation of these polymers may yield low-molecular-weight components that can participate in Maillard reactions and may exhibit enhanced antioxidant activity.

A particularly promising application of this approach involves by-products of the meat and poultry industries, which are rich in both collagen and GAGs derived from cartilaginous tissues. In such systems, the sequential combination of enzymatic protein hydrolysis with GAGs degradation may facilitate the formation of Maillard reaction products that contribute to flavor development while simultaneously providing antioxidant protection in food products.

## Figures and Tables

**Table 1:** Amino acid composition of collagen and hydrolysates [residues/100 amino acid residues *].

Amino Acids	ColTKC	HP	HMIX
Asp	6.81 ± 0.09	6.05 ± 0.20	6.12 ± 0.95
Thr	2.37 ± 0.10	2.04 ± 0.36	2.13 ± 0.51
Ser	2.87 ± 0.45	2.46 ± 0.75	2.54 ± 0.56
Glu	10.06 ± 0.25	8.90 ± 0.96	8.39 ± 0.82
Pro	9.69 ± 0.70	9.45 ± 0.90	9.55 ± 0.66
Gly	25.45 ± 0.20 ^b^	31.82 ± 0.58 ^a^	30.90 ± 1.27 ^a^
Ala	5.64 ± 0.50 ^b^	5.60 ± 0.17 ^b^	6.98 ± 0.42 ^a^
Cys	0.13 ± 0.04 ^a^	0.06 ± 0.01 ^b^	0.09 ± 0.02 ^a,b^
Val	2.29 ± 0.76	1.94 ± 0.40	2.18 ± 0.83
Met	1.01 ± 0.07 ^a^	0.86 ± 0.03 ^b^	0.83 ± 0.09 ^b^
Ile	1.91 ± 0.11	1.59 ± 0.17	1.65 ± 0.19
Leu	3.56 ± 0.25	2.98 ± 0.12	3.12 ± 0.29
Tyr	0.46 ± 0.05 ^b^	0.43 ± 0.08 ^b^	0.68 ± 0.09 ^a^
Phe	2.36 ± 0.18	1.98 ± 0.58	2.16 ± 0.21
His	0.73 ± 0.03 ^b^	0.54 ± 0.17 ^b^	1.50 ± 0.25 ^a^
Lys	3.08 ± 0.25	2.53 ± 0.61	2.70 ± 0.80
Arg	8.36 ± 0.75	7.04 ± 0.42	6.62 ± 0.85
HPro	13.22 ± 0.69	13.73 ± 0.83	11.86 ± 0.90
HPro to collagen conversion factor [dimensionless]	7.56	7.28	8.43

(*n* = 3); ColTKC—reconstituted atelocollagen fibers from turkey knee cartilage; HP—Protamex hydrolysate; HMIX—enzymatic hydrolysates obtained with the three-enzyme mixture (papain, Protamex and Flavourzyme). Letters a, b indicate significant differences between samples (*p* < 0.05). * The unit [residues/100 amino acid residues] corresponds to the percentage share of a given amino acid in the total amino acid content.

**Table 2:** Color parameters of collagen, hydrolysates and Maillard reaction products.

Sample				Color Parameters		
				L*	a*	b*
ColTKC				81.69 ± 0.57 ^b,A^	−1.69 ± 0.03 ^g,H^	10.99 ± 0.14 ^d,E^
HP				90.79 ± 0.26 ^a^	−0.94 ± 0.08 ^g^	16.93 ± 0.53 ^b,c^
MRPs	HP	60 °C	CS-S	77.02 ± 1.36 ^c^	−0.80 ± 0.13 ^g^	23.17 ± 0.05 ^a^
			CS-T	76.18 ± 1.10 ^c,d^	4.05 ± 0.07 ^d,e^	20.99 ± 0.16 ^a^
		80 °C	CS-S	73.80 ± 1.58 ^d,e^	1.59 ± 0.35 ^f^	22.73 ± 1.16 ^a^
			CS-T	73.23 ± 0.66 ^d,e^	3.70 ± 0.46 ^e^	23.13 ± 0.57 ^a^
		100 °C	CS-S	66.29 ± 2.72 ^f^	5.58 ± 0.90 ^b,c^	23.05 ± 1.16 ^a^
			CS-T	72.80 ± 0.64 ^e^	3.02 ± 0.76 ^e^	21.99 ± 0.01 ^a^
		120 °C	GM	54.70 ± 2.57 ^g^	6.42 ± 1.13 ^a,b^	14.47 ± 3.73 ^c^
			CS-S	54.70 ± 0.13 ^g^	7.47 ± 0.09 ^a^	18.22 ± 0.31 ^b^
			CS-T	64.76 ± 0.33 ^f^	5.06 ± 0.62 ^c,d^	21.87 ± 0.01 ^a^
HMIX				77.17 ± 0.17 ^B^	2.83 ± 0.12 ^E,F^	23.47 ± 0.12 ^A,B^
MRPs	HMIX	60 °C	CS-S	73.53 ± 0.33 ^C,D^	4.08 ± 0.06 ^D,E^	25.43 ± 0.33 ^A^
			CS-T	73.11 ± 0.49 ^C,D^	5.96 ± 0.28 ^B,C^	19.22 ± 0.76 ^C,D^
		80 °C	CS-S	72.40 ± 0.13 ^D^	5.10 ± 0.13 ^C,D^	24.87 ± 0.35 ^A^
			CS-T	76.19 ± 0.56 ^B,C^	3.09 ± 0.88 ^E,F^	18.40 ± 0.04 ^D^
		100 °C	GM	42.24 ± 1.05 ^G^	7.82 ± 1.45 ^A^	8.48 ± 2.74 ^F^
			CS-S	64.50 ± 0.15 ^E^	5.60 ± 0.38 ^C^	20.45 ± 0.82 ^C,D^
			CS-T	73.80 ± 0.81 ^C,D^	2.46 ± 0.09 ^F,G^	18.63 ± 0.59 ^D^
		120 °C	GM	30.55 ± 0.78 ^H^	1.48 ± 0.05 ^G^	1.20 ± 0.14 ^G^
			CS-S	60.19 ± 0.60 ^F^	7.16 ± 0.85 ^A,B^	21.31 ± 0.69 ^B,C^
			CS-T	65.90 ± 0.54 ^E^	5.35 ± 0.28 ^C,D^	19.47 ± 0.21 ^C,D^

(*n* = 3); ColTKC—reconstituted atelocollagen fibers from turkey knee cartilage; HP—Protamex hydrolysate; HMIX—enzymatic hydrolysate obtained with the three-enzyme mixture (papain, Protamex and Flavourzyme); MRPs—Maillard reaction products; GM—glucosamine; CS-S—shark chondroitin sulfate hydrolysate; CS-T—turkey chondroitin sulfate hydrolysate. Capital letters in the columns indicate significant differences between samples for HMIX, lowercase letters for HP (*p* < 0.05).

**Table 3:** Antioxidant activity (ABTS) and ferric reducing antioxidant power (FRAP) of collagen hydrolysates and Maillard reaction products.

Sample				ABTS [%]	FRAP [Fe^2+^ mM/g]
HP				25.43 ± 1.20 ^k^	20.50 ± 5.25 ^l^
MRPs	HP	60 °C	G	33.86 ± 2.17 ^i^	28.67 ± 6.30 ^l^
			GM	64.86 ± 1.27 ^d,e^	178.94 ± 8.70 ^e^
			CS-S	30.57 ± 0.72 ^j^	72.59 ± 3.38 ^j^
			CS-T	56.58 ± 1.00 ^f^	180.80 ± 6.29 ^e^
		80 °C	G	32.86 ± 2.65 ^i,j^	28.96 ± 6.84 ^l^
			GM	67.00 ± 0.52 ^c,d^	197.80 ± 762 ^d^
			CS-S	34.57 ± 0.53 ^i^	90.29 ± 5.18 ^i^
			CS-T	57.29 ± 0.96 ^f^	141.85 ± 1.00 ^g^
		100 °C	G	33.86 ± 0.86 ^i^	40.17 ± 1.92 ^k^
			GM	78.43 ± 1.37 ^b^	332.67 ± 8.66 ^a^
			CS-S	47.43 ± 2.32 ^g^	161.21 ± 6.22 ^f^
			CS-T	62.71 ± 2.63 ^e^	130.52 ± 8.48 ^h^
		120 °C	G	39.43 ± 1.20 ^h^	63.07 ± 3.08 ^j^
			GM	81.00 ± 1.00 ^a^	335.99 ± 5.77 ^a^
			CS-S	76.14 ± 1.15 ^b^	287.32 ± 6.13 ^b^
			CS-T	67.86 ± 0.99 ^c^	271.88 ± 1.59 ^c^
HMIX				29.86 ± 1.53 ^J^	45.93 ± 5.69 ^L^
MRPs	HMIX	60 °C	G	34.29 ± 1.30 ^I^	43.01 ± 2.01 ^L^
			GM	55.71 ± 1.01 ^F^	166.38 ± 4.27 ^G^
			CS-S	37.14 ± 1.72 ^H^	142.47 ± 2.45 ^I^
			CS-T	58.29 ± 2.77 ^E^	150.29 ± 4.97 ^H^
		80 °C	G	38.14 ± 2.74 ^H^	49.94 ± 5.36 ^L^
			GM	65.00 ± 1.11 ^D^	238.19 ± 1.11 ^E^
			CS-S	38.43 ± 0.43 ^H^	129.51 ± 1.38 ^J^
			CS-T	58.42 ± 0.66 ^E^	164.77 ± 3.82 ^G^
		100 °C	G	38.14 ± 0.90 ^H^	70.01 ± 1.47 ^K^
			GM	73.29 ± 0.28 ^B^	348.59 ± 4.18 ^B^
			CS-S	50.57 ± 0.57 ^G^	208.70 ± 8.37 ^F^
			CS-T	69.43 ± 0.54 ^C^	260.98 ± 5.21 ^D^
		120 °C	G	50.29 ± 1.72 ^G^	144.07 ± 1.99 ^H,I^
			GM	80.13 ± 1.20 ^A^	379.49 ± 2.24 ^A^
			CS-S	69.14 ± 1.00 ^C^	290.50 ± 2.37 ^C^
			CS-T	63.43 ± 1.43 ^D^	285.91 ± 5.80 ^C^

(*n* = 3); HP—Protamex hydrolysate; HMIX—enzymatic hydrolysate obtained with the three-enzyme mixture (papain, Protamex and Flavourzyme); MRPs—Maillard reaction products; G—glucose; GM—glucosamine; CS-S—shark chondroitin sulfate hydrolysate; CS-T—turkey chondroitin sulfate hydrolysate. Capital letters in the columns indicate significant differences between samples for HMIX, lowercase letters for HP (*p* < 0.05).

## Data Availability

Data are available from the corresponding author.
